# The *JHAMT1* gene is necessary for wing differentiation in *Aphis gossypii*

**DOI:** 10.3389/fphys.2026.1747260

**Published:** 2026-03-10

**Authors:** Jingli Lv, Liuyu Wang, Xiangzhen Zhu, Li Wang, Meishuang Jian, Kaixin Zhang, Dongyang Li, Xueke Gao, Jichao Ji, Junyu Luo

**Affiliations:** 1 Research Base of Zhengzhou University, State Key Laboratory of Cotton Bio-Breeding and Integrated Utilization, Institute of Cotton Research, Chinese Academy of Agricultural Sciences, Anyang, Henan, China; 2 Western Agricultural Research Center, Chinese Academy of Agricultural Sciences, Changji, China; 3 State Key Laboratory of Cotton Bio-Breeding and Integrated Utilization, School of Agricultural Sciences, Zhengzhou University, Zhengzhou, Henan, China

**Keywords:** *Aphis gossypii*, juvenile hormone acid methyltransferase, RNAi, temporal transcriptome analysis, wing differentiation

## Abstract

**Background:**

*Aphis gossypii* (cotton–melon aphid) is ranked among the top 10 most destructive agricultural pests worldwide, inflicting substantial damage on a wide range of host plants annually. Wing polyphenism is a key trait that facilitates rapid population expansion and efficient virus transmission. However, the molecular mechanisms underlying wing differentiation in this sap-sucking pest remain poorly understood.

**Results:**

The differentiation dynamics of newborn *A*. *gossypii* nymphs into to alate (winged) or apterous (wingless) morphs was characterized, coupled with pairwise morphological comparisons. The second- and third-instar nymphal stages were identified as two critical turning points for wing morph determination. The number of differentially expressed genes between alate and apterous morphs increased during development, peaking at the fourth-instar nymphal stage before declining in adulthood. Notably, compared with that in the apterous morph, upregulated genes in the alate morph at each developmental stage were consistently enriched in the juvenile hormone (JH) biosynthesis signaling pathway. Four *JHAMT* (juvenile hormone acid methyltransferase)—encoding the rate-limiting enzyme in JH biosynthesis—were identified in the *A. gossypii* genome. Among these, only *JHAMT*1 exhibited significantly elevated expression in the second- and third-instar nymphs of the alate morph, corresponding to the critical period of wing differentiation. Knockdown of *JHAMT*1 via ds*JHAMT*1 feeding in third-instar alate nymphs resulted in a 79% reduction in transcript level and significantly inhibited normal wing differentiation in 60.2% of the treated individuals. Notably, wing buds of these affected nymphs failed to develop and eventually disappeared in adulthood, demonstrating that *JHAMT1* is indispensable for wing morph formation in *A. gossypii*.

**Conclusion:**

This study clarifies the complete differentiation process of *A. gossypii* into alate or apterous morphs and confirms that *JHAMT*1, a key regulatory gene in JH biosynthesis, plays a pivotal role in wing differentiation of the alate morph. Given its essential function in wing development, *JHAMT1* holds strong potential as a molecular target for developing strategies to control outbreaks and spread of the cotton aphid.

## Hightlights

1. Wing morphs in *A. gossypii* can also be post-embryonically induced rather than being solely dependent on maternal transmission.
2. The dynamics of wing differentiation in *A. gossypii* were clearly delineated, with gene expression exhibiting a peak-like pattern and the JH synthesis pathway being enriched throughout all the stages of wing development.
3. Four *JHAMT* genes were identified in *A. gossypii*, of which only *JHAMT*1 is highly expressed during the critical stage of wing differentiation, and it is located on the opposite strand of the genome compared to the other three *JHAMT* genes.
4. *JHAMT*1 is involved in the wing differentiation of *A. gossypii*, whereas the functional roles of the remaining *JHAMT* homologs remain to be elucidated.

## 1 Introduction

*Aphis gossypii* Glover (Hemiptera: Aphididae), commonly referred to as the cotton–melon aphid, is a polyphagous agricultural pest with a global distribution spanning over 171 countries (Wills, 2017). As one of the top ten most destructive agricultural pests, it infests a diverse range of crops, including those belonging to the families Cucurbitaceae, Solanaceae, Leguminosae, and Asteraceae (Huangfu et al., 2022; Ji et al., 2021). By directly or indirectly sucking the plant sap, transmitting plant viruses, and secreting honeydew, this pest causes significant losses in crop yield and quality annually (Heilsnis et al., 2023; Elbanhawy et al., 2019).

The enormous economic losses caused by aphids (including *A. gossypii*) to agricultural systems are closely associated with their remarkable phenotypic plasticity, particularly the capacity to switch between wing differentiation and rapid reproduction. Specifically, aphids adopt alate (winged) or apterous (wingless) morphs in response to environmental cues, such as host nutrition status, population density, and natural enemy pressure (Guo et al., 2023). The apterous morph, endowed with high fecundity, reproduces predominantly via parthenogenesis (Braendle et al., 2006), which facilitates rapid colonization and population expansion under favorable habitat conditions, thereby inflicting severe damage to crops, fruits, seedlings, and other vegetation (Simon et al., 2018; Wang et al., 2015). Once environmental conditions deteriorate, aphids undergo an apterous morph and switch to the alate form, migrating to new host plants and establishing fresh infestations (Tsuji and Kawada, 1987). Wing dimorphism constitutes a key adaptive strategy for aphids to cope with fluctuating environments and plays a pivotal role in extending their distribution and promoting the transmission of aphid-borne viruses (Tsumuki et al., 1990). However, the molecular mechanisms governing wing differentiation in this sap-sucking hemipteran remain inadequately elucidated.

Currently, the management of *A. gossypii* still relies predominantly on chemical insecticides, particularly neonicotinoids such as imidacloprid, acetamiprid, and thiamethoxam (Eid et al., 2018). These insecticides exhibit high efficacy and broad-spectrum activity, exerting strong insecticidal effects against a wide range of pest species (Shi et al., 2023). Nevertheless, long-term over-reliance on chemical insecticides has led to the rapid evolution of resistance in *A. gossypii*, a significant decline in beneficial natural enemies in agricultural fields, compromised biological control efficacy, and, ultimately, induced aphid resurgence (Cheng et al., 2023; Li et al., 2022). Therefore, there is an urgent need to develop new control strategies for the management of *A. gossypii*. Elucidating the molecular mechanism underlying wing differentiation in *A. gossypii* assists in developing genetic-based control strategies against this pest by disrupting its flight behavior through the application of dsRNA-based pesticides or the planting of transgenic crops. Wing polyphenism is a prevalent adaptive strategy in insects, evolving in response to complex environmental conditions and serving as a key factor of population expansion. In aphids, populations comprise both alate (flight-capable) and apterous (flight-incapable) morphs, with wing morph determination largely depending on transgenerational signal transduction (Brisson, 2010; Ogawa and Miura, 2014). Extensive studies on aphid wing morph transition have focused on several species, revealing multiple regulatory pathways. Ecdysone signaling was the first pathway implicated in aphid wing polyphenism (Vellichirammal et al., 2017). In *Acyrthosiphon pisum*, both winged and wingless individuals have organized wing primordia within 24 h after birth, but the wing primordia of wingless individuals undergo programmed degeneration within prominent autophagy 36 h later. Ecdysone or analog injection reduced alate offspring production, while ecdysone signaling disruption—via receptor antagonists or gene knockdown—increased alate offspring (Vellichirammal et al., 2017).

Subsequently, TORC1 (eukaryotic target of rapamycin complex 1 kinase), a key regulator of wing disc size, was identified in *Drosophila* (Strassburger et al., 2021), and its downstream transcription factor REPTOR2 was identified as a critical modulator. Overexpression of REPTOR2 promotes autophagy of wing primordia, thereby reducing the proportion of alate individuals (Yuan et al., 2023). In contrast, the insulin signaling (INS) pathway regulates wing differentiation in *Aphis citricidus*, where silencing the insulin receptor genes in the fourth-instar alate nymphs induces adult wing malformation, and miR-9b targets the ABC (ATP-binding cassette) transporter ABCG4 to modulate the INS pathway activity, thereby regulating wing dimorphism (Shang et al., 2020).

Compared to the ecdysone, TOR, and INS pathways, the roles of the juvenile hormone (JH) signaling pathway in insect wing differentiation and development have also received considerable attention but remain controversial. In *Megoura crassicauda*, wing differentiation correlates closely with JH titers: JH III titers are significantly higher in wingless than in winged morphs, and the application of JH III to third-instar winged nymphs significantly inhibited wing differentiation, resulting in winged/wingless intermediates and wingless ultra-larvae with an extra molting (Ishikawa et al., 2013). However, no clear association was found between maternal JH titers and the production of winged progeny, indicating that aphid wing dimorphism may be regulated by alternative physiological mechanisms (Schwartzberg et al., 2008).

Our previous work demonstrated that postnatal crowding alone induces extensive winged morph differentiation in newborn *A. gossypii*. Transcriptomic analysis revealed that upregulated genes in alate morphs were enriched in the JH biosynthesis pathway, with the key regulatory gene *JHAMT* showing a 7.14-fold upregulation—implying that JH is a potential key regulator of wing differentiation in this species (Ji et al., 2019). Most aphids synthesize two JH isoforms (JH III and JH SB3), with JH III predominating. Unlike the canonical FA–MF–JH III synthesis pathway in most insects, aphids adopt an FA–JHA–JH III pathway, involving epoxidation by CYP15C1 followed by methylation by *JHAMT* (Li et al., 2025). As a key enzyme regulating JH titers, *JHAMT* is well-documented to modulate the development, metamorphosis, and reproduction in various insects. In *Tribolium castaneum*, inhibition of the *JHAMT* via an antagonist in larvae led to molting failure, pupal melanization, and a drastic decline in survival rate (Yang et al., 2026). In *Apis mellifera*, the downregulation of *JHAMT* expression via ame-miR-2161 decreased larval survival rates and body weight (Song et al., 2026). However, its role in regulating wing differentiation remains poorly understood.

In this study, we elucidated the differentiation dynamic of *A. gossypii* into alate and apterous morphs and systematically performed pairwise comparisons of the two morphs across all differentiation stage. RNA-seq profiling uncovered stage-specific variations in gene expression between the alate and apterous morphs of *A. gossypii*, indicating that the JH biosynthetic signaling pathway likely mediates the development of the alate morph in this pest. Furthermore, RNA interference validated *JHAMT1*—the key regulatory gene of JH biosynthesis—as a critical determinant of wing differentiation in this aphid species. Collectively, our findings advance the theoretical understanding of aphid wing differentiation and provide novel insights for studying insect wing dimorphism.

## 2 Methods and materials

### 2.1 Insects and plants

*A. gossypii* was reared and purified for multiple generations in the Laboratory of the Institute of Cotton Research, Chinese Academy of Agricultural Sciences (Anyang, Henan Province, China). Cotton seedlings (CCRI 49) were cultured at 25 °C ± 1 °C and 75% ± 5% relative humidity and under a 14 h light/10 h dark photoperiod. Apterous and alate morphs were induced by manipulating population density following previous protocols (Ji et al., 2019), and the experimental schematic is presented in Supplementary Figure S1.

### 2.2 Morphological characteristics and sample collection

The morphological dynamics of newborn nymphs differentiating into apterous or alate morphs—especially the wing buds or wing sacs on the prothorax and mesothorax at each developmental stage—were recorded and measured using a SteREO Discovery V8 microscope (Zeiss, Germany). Correspondingly, whole bodies of apterous and alate individuals were collected at each differentiation stage (second instar to adult) during the wing differentiation process or programmed degeneration of wing primordia, respectively. Specifically, apterous and alate individuals were sampled at each stage (second instar to adult) 12 h post-molting. Three biological replicates were prepared for each stage of each morph, with ≥50 individuals per replicate; notably, both the first and second instars contained ≥200 individuals. A total of 27 samples were obtained, immediately frozen in liquid nitrogen, and then transferred to a −80 °C freezer for storage.

### 2.3 Transcriptome assembly and annotation

Total RNA was extracted using the RNAiso Plus kit (TaKaRa, Japan), following the manufacturer’s instructions. After quality verification, cDNA libraries were constructed and sequenced on the Illumina NovaSeq 6000 platform. Raw reads generated from sequencing were filtered to remove adaptor sequences and low-quality reads, yielding clean reads. Clean RNA-seq reads were aligned to the *A. gossypii* genome (version ASM2018417v2) using HISAT2 (Kim et al., 2015; Zhang et al., 2022). Transcripts for each sample were reconstructed with StringTie and compared against the original genome annotation (Pertea et al., 2015). Novel genes were subsequently aligned to the NCBI non-redundant (NR), Swiss-Prot, COG (Cluster of Orthologous Groups), KOG (Clusters of Orthologous Groups for Eukaryotic Complete Genomes), and KEGG (Kyoto Encyclopedia of Genes and Genomes) databases using DIAMOND (Buchfink et al., 2015), while GO (Gene Orthology) functional annotation was performed via InterProScan (Jones et al., 2014).

### 2.4 Identification and functional analysis of differentially expressed genes

The gene expression levels of each sample were calculated using StringTie and normalized as fragments per kilobase of transcript per million mapped reads (FPKM) (Pertea et al., 2015). DESeq2 was utilized to identify differentially expressed genes (DEGs) between the apterous and alate morphs at each pairwise development stage, namely, the second-instar apterous nymph vs. the second-instar alate nymph (WL–N2 vs. W–N2), the third-instar apterous nymph vs. the third-instar alate nymph (WL–N3 vs. W–N3), the fourth-instar apterous nymph vs. the fourth-instar alate nymph (WL–N4 vs. W–N4), and the apterous adult vs. alate adult (WL–A vs. W–A). The threshold for significant DEGs was set as |log 2 FC (fold change) | ≥ 2 with a false discovery rate (FDR) < 0.05. KEGG pathway enrichment analyses of DEGs were performed using the hypergeometric test (threshold: q-value <0.05) to explore their potential biological functions with a threshold of q-value <0.05.

### 2.5 Quantitative real-time PCR validation

To verify the reliability of the transcriptome data, the relative expression levels of nine selected genes were quantified via RT-qPCR using a LightCycler 480 instrument (Roche Diagnostics, Switzerland). The 20-μL quantitative real-time PCR (RT-qPCR) system consisted of 2 μL cDNA template, 7.2 μL nuclease-free water, 0.4 μL each of forward/reverse primers, and 10 μL 2× TransStart® Top Green qPCR SuperMix (+DyeI/+DyeII) (TransGen Biotech, Cat. No. AQ131). The thermal cycling program was set as follows: pre-denaturation at 95 °C for 5 min, followed by 40 cycles of 95 °C for 10 s, 60 °C for 10 s, and 72 °C for 10 s. Gene-specific primers were designed with Primer Premier 6 and synthesized by Shanghai Sangon Biotech Co., Ltd. Additionally, the *EF1α* gene served as an internal reference to normalize the gene expression levels (Ma et al., 2016). All primers were verified for specificity by alignment with the *A. gossypii* genome and melting curve analysis, and their sequences are listed in Supplementary Table S1. The relative gene expression levels were calculated using the 2^−ΔΔCt^ method (Livak and Schmittgen, 2001).

### 2.6 dsRNA synthesization and RNAi assays

Specific fragments of the RNAi target gene *(JHAMT*1) were amplified by PCR using T7 promoter sequences, and double-stranded RNA (dsRNA) was synthesized and purified *in vitro* using the T7 RNAi Transcription Kit (Vazyme, China). RNAi assays were conducted on the third-instar alate nymphs using a custom feeding system: a glass tube with Parafilm layers sealed at both ends. A 30% sucrose solution containing 1 μg/μL dsRNA was sandwiched between the Parafilm layers at the top, and third-instar alate nymphs that had molted within 12 h were gently transferred into the apparatus. Three biological replicates were set for each group, with 50 individuals per replicate. The glass tubes were incubated at 25 °C ± 1 °C under a 14 h light/10 h dark photoperiod in an incubation chamber. A trace amount of green pigment was added to the sucrose solution prior to the experiment; individuals with the green pigment visible under the body wall were considered to have ingested the dsRNA-containing diet. RNAi efficacy was evaluated via RT-qPCR 48 h post-feeding. The dsRNA-containing sucrose solution was replaced every 3 days, and the numbers of alate and apterous morphs were counted on day 6. The relative gene expression was calculated according to the 2^−ΔΔCt^ method (Livak and Schmittgen, 2001). Double-stranded green fluorescent protein (dsGFP) was used as a control, and all experimental procedures were identical to those used for the treatment group, as described above.

### 2.7 Statistical analysis

One-way analysis of variance (ANOVA), Duncan’s new multiple-range test, and t-test were used to analyze the differences among the experimental groups, with a significance level set at *p* < 0.05.

## 3 Results

### 3.1 Morphological differences between apterous and alate morphs during development

**FIGURE 1:**
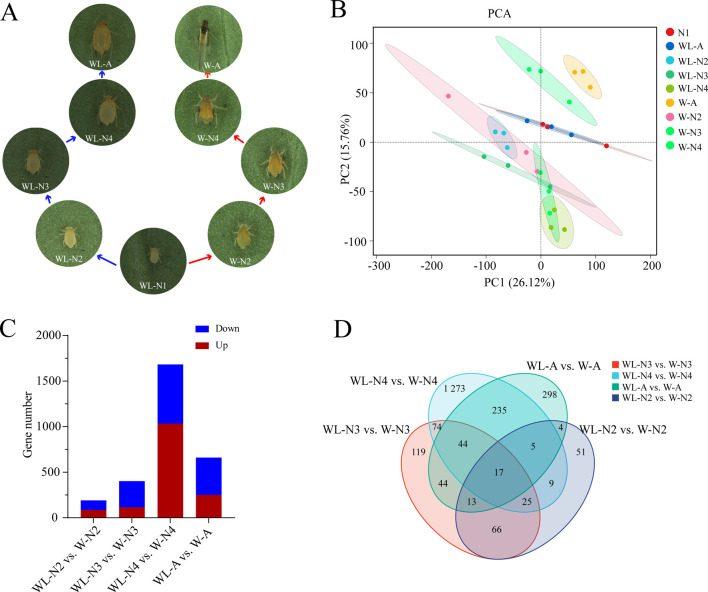
Comparison of gene expression dynamics between alate and apterous morphs of cotton aphid during development and differentiation. **(A)** Morphological dynamics of apterous and alate morphs differentiated from the first nymph under low (left) or high (right) population densities, respectively. **(B)** PCA of transcriptome samples from apterous and alate morphs across their respective developmental process. **(C)** Statistics of DEGs from pairwise comparison: WL–N2 vs. W–N2, WL–N3 vs. W–N3, WL–N4 vs. W–N4, and WL–A vs. W–A. **(D)** Venn diagram showing the distribution of DEGs from pairwise comparisons at each development stage between the two morphs. Abbreviations: WL–N2, WL–N3, WL–N4, WL–A, W–N2, W–N3, W–N4, and W–A represent wingless (WL) and wing (W) morphs at the second-instar nymph (N2), third-instar nymph (N3), fourth-instar nymph (N4), and adult **(A)** stages, respectively.

Compared with apterous morphs, the alate morphs induced by rearing newborn nymphs under high population density underwent four developmental stages: wing primordium differentiation, wing sac formation, wing bud formation, and full wing eclosion (Figure 1A). No obvious morphological differences were observed between the first-instar nymphs reared under crowded and low-density conditions, which is attributed to their short developmental duration. Specifically, the second-instar alate and apterous nymphs were morphologically highly similar; however, a slight bulge of the wing primordium was present on the anterior edge of the thorax in alate nymphs, indicating ongoing wing primordium differentiation. Distinct morphological differences between alate and apterous morphs became distinguishable from the third-instar nymph stage, with alate nymphs completing wing capsule formation—marking accelerated wing differentiation (Figure 1A; Supplementary Figure S2). Compared to apterous nymphs, the fourth-instar alate nymphs exhibited distinct wing buds on both sides of the thorax, and the wings were fully expanded upon adult emergence. Overall, the wing differentiation process of alate morphs can be divided into four stages (Supplementary Figure S2), namely, the preliminary stage (second-instar nymph), prophase (third-instar nymph), acceleration stage (fourth-instar nymph), and emergence period (adult).

### 3.2 Transcriptome data overview and sample clustering analysis

Gene expression levels at four differentiation stages (second, third, fourth, and adult) were subjected to pairwise comparisons between apterous and alate morphs. A total of 186.38 Gb of clean reads were generated, with a Q30 quality score ≥89.46% and a mapping ratio of >95.62% against the *A. gossypii* genome (ASM2018417v2) (Supplementary Table S2). Principal component analysis (PCA) results revealed that the intergroup sample distance was greater than the intragroup distance, with low variability among replicates of the same stage (Figure 1B). Furthermore, compared to the distinct separation of the fourth/adult alate morphs from other samples, the correlation between the apterous and alate samples at the first, second, and third instars was relatively higher (Figure 1B). This observation was consistent with the dynamic morphological characteristic changes during wing differentiation (Figure 1A), where significant differences in wing development emerged from the fourth-instar stage onward.

With the wing differentiation, the number of DEGs between the alate and apterous wing morphs at each developmental stage gradually increased from 164 to 1,678 (Figure 1C). Notably, compared to apterous morphs, the number of significantly upregulated genes in alate morphs peaked at 1,030 in the fourth-instar nymphs, indicating that a large number of essential genes require enhanced expression during this critical wing-bud formation stage of wing differentiation. Notably, compared to the apterous morph, the number of downregulated genes in the wing morph (88, 260, and 406, respectively) was significantly higher than that of upregulated genes (76, 96, and 249, respectively) in the second-instar nymph, third-instar nymph, and adult stages. Additionally, 17 shared DEGs were identified across all the development stages between the two morphs, while the number of stage-specific DEGs between them was 51, 119, 1,273, and 298 for each stage, respectively (Figure 1D).

### 3.3 Functional analysis of DEGs between alate and apterous morphs

**FIGURE 2:**
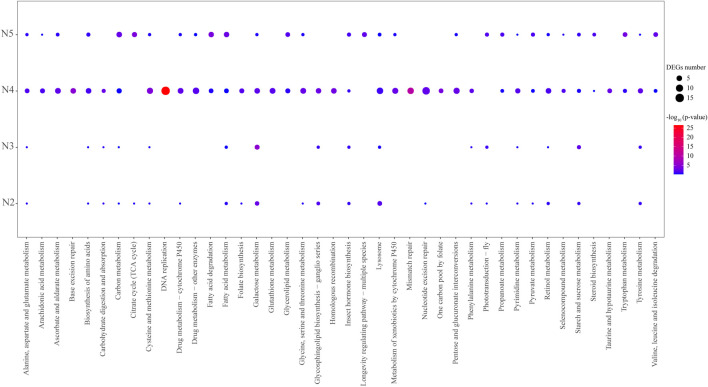
KEGG pathway enrichment analysis of significantly upregulated DEGs from pairwise comparisons of WL–N2 vs. W–N2, WL–N3 vs. W–N3, WL–N4 vs. W–N4, and WL–A vs. W–A. Significantly enriched pathways were identified using the hypergeometric test with an adjusted *p-*value <0.05.

**FIGURE 3:**
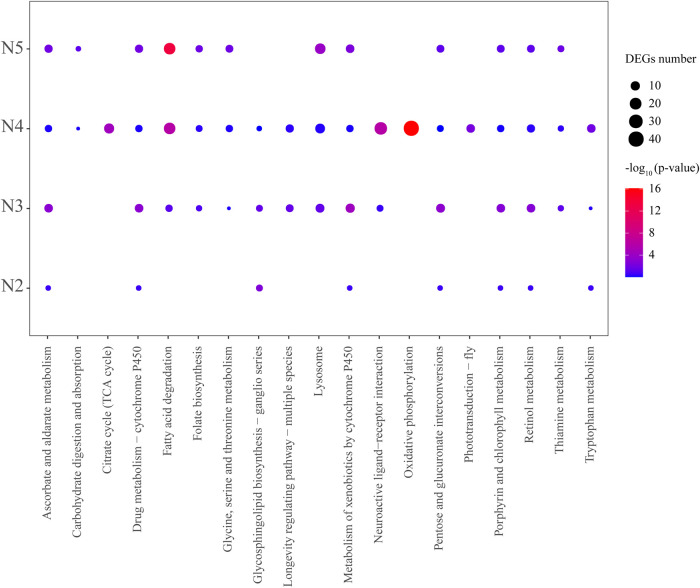
KEGG pathway enrichment analysis of significantly downregulated DEGs in pairwise comparison of WL–N2 vs. W–N2, WL–N3 vs. W–N3, WL–N4 vs. W–N4, and WL–A vs. W–A. Significantly enriched pathways were identified using the hypergeometric test with an adjusted *p*-value <0.05

To explore the potential signaling pathways and vital genes involved in the wing formation regulation, the upregulated and downregulated DEGs between the alate and apterous morphs at each developmental stage were analyzed via KEGG enrichment, respectively. Overall, those DEGs were mainly enriched in signaling pathways related to metabolism (i.e., carbohydrate metabolism, amino acid metabolism, metabolism of cofactors and vitamins, lipid metabolism, and metabolism of terpenoids and polyketides), genetic information processing (i.e., replication and repair, signaling molecules, and interaction), organismal systems (i.e., aging, sensory system, and digestive system), environmental information processing (i.e., signaling molecules and interaction), and cellular processes (i.e., transport and catabolism) (Figures 2, 3; Supplementary Tables S3 and S4).

In WL–N2 vs. W–N2, upregulated DEGs in W–N2 were significantly enriched in lysosome, galactose metabolism, and glycosphingolipid biosynthesis (Figure 2), while downregulated DEGs were only enriched in glycosphingolipid biosynthesis (Figure 3). For WL–N3 vs. W–N3, upregulated DEGs in W–N3 were significantly enriched in starch and sucrose metabolism and galactose metabolism (Figure 2), whereas the downregulated DEGs were primarily enriched in pathways including longevity regulation, pentose and glucuronate interconversions, ascorbate and aldarate metabolism, porphyrin and chlorophyll metabolism, retinol metabolism, and thiamine metabolism (Figure 3).

In WL–N4 vs. W–N4, upregulated DEGs in alate morphs were significantly enriched in 27 pathways, such as DNA replication, galactose metabolism, biosynthesis of amino acids, retinol metabolism, and phenylalanine metabolism (Figure 2), whereas the downregulated DEGs were mostly enriched in oxidative phosphorylation, neuroactive ligand–receptor interaction, citrate cycle (TCA cycle), fatty acid degradation, and phototransduction (Figure 3). For WL–A vs. W–A, upregulated DEGs in alate morphs were significantly enriched in 13 pathways, such as longevity regulation, fatty acid degradation, tryptophan metabolism, the citrate cycle (TCA cycle), and insect hormone biosynthesis (Figure 2), whereas downregulated DEGs were mostly enriched in 12 pathways, including fatty acid degradation, lysosomes, ascorbate and aldarate metabolism, and folate biosynthesis (Figure 3).Notably, galactose metabolism was enriched in three development stages (second- to fourth-instar nymphs) in the comparison between the two morphs. A total of 11 pathways were identified through enrichment analysis of upregulated genes across all groups, including starch and sucrose metabolism, tyrosine metabolism, and galactose metabolism. Among them, insect hormone (juvenile hormone and 20E) biosynthesis drew our attention, as DEGs in this signaling pathway were detected across all four development stages during wing morph differentiation (Figures 2, 3).

Additionally, our previous study demonstrated that postnatal crowding alone induces numerous newborn nymphs to differentiate into alate morphs in cotton aphids, and upregulated genes in alate morphs were also enriched in the JH biosynthesis signaling pathway (Ji et al., 2019), indicating that JH may be a key regulator of wing differentiation in cotton aphids. Specifically, four juvenile hormone acid O-methyltransferase genes (*JHAMT*)—encoding a critical enzyme that converts JH acids or inactive precursors to active JHs in the final step of insect JH biosynthesis—were identified in the transcriptome. However, only *LOC114124070* (designated *JHAMT1*) exhibited significantly elevated expression levels during two key wing-differentiation periods: 20.23- and 6.33- fold higher than in apterous morphs at the second- and third-instar nymph stages, respectively. Its expression subsequently declined, even reaching one-fourth of that in apterous morphs in adulthood (Supplementary Figure S4). Taken together, these findings highlight the importance of *JHAMT1* (*LOC114124070*) in regulating wing morph differentiation of *A. gossypii*.

### 3.4 *JHAMT* gene distribution in *Aphis gossypii*

**FIGURE 4:**
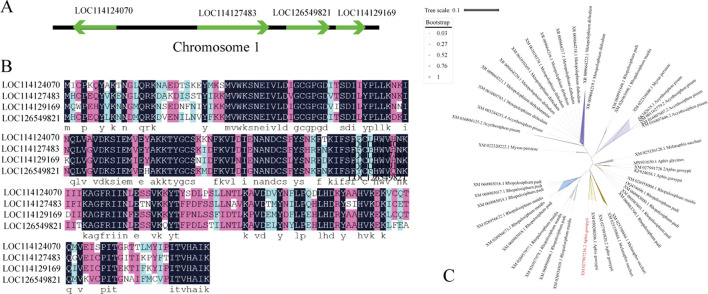
*JHAMT*s in several aphid species. **(A)** Distribution of four *JHAMT* genes on chromosome 1 of *A. gossypii* genome. **(B)** Multiple sequence alignment of the coding sequences of four *JHAMT* genes in *A. gossypii*. **(C)** Phylogenetic tree of *JHAMT* genes from several aphid species.

To explore the role of *JHAMT* in wing differentiation of *A. gossypii*, the reliability of RNA-seq data was validated via qPCR, in which nine DEGs—including *CTSB* (cathepsin B), *NIT2* (omega-amidase), *EIP93F*, *JHAMT*, and HPGD—were selected. The results showed that the expression trends of those qPCR-validated DEGs across pairwise comparisons of the four development stages between the apterous and alate morphs were consistent with those from the transcriptome (Supplementary Figure S3), confirming the reliability of our RNA-seq data. In the *A. gossypii* genome, four *JHAMT* genes (*LOC114124070*, *LOC114127483*, *LOC126549821*, and *LOC114129169*) were all located on the same strand of chromosome 1, except *LOC114124070*, which was located on the opposite strand. Their lengths are 1,992 bp, 5,662 bp, 3,418 bp, and 2,338 bp, respectively. The genetic distances between these *JHAMT* genes range from 8.2 kb to 39.17 Mb, with *LOC114124070* being significantly distant from the other three (Figure 4A).

In our transcriptome, the full-length transcript sequence identity among the four *JHAMT* genes was only 37.13%, whereas their coding sequence (CDS) identity reached 91.85%. These CDSs encode separate protein sequences of 266 amino acid residues, with 84.77% sequence identity (Figure 4B). Notably, many insect species possess multiple *JHAMT* genes. We retrieved *JHAMT* genes from several aphid species from NCBI and constructed a phylogenetic tree. The results showed that *JHAMT* genes from the same species typically clustered closely, forming four major clades corresponding to *Metopolophium dirhodum*, *A*. *pisum*, *Rhopalosiphum maidis*, and *Rhopalosiphum padi* (Figure 4C). Interestingly, *JHAMT* genes from some aphid species (i.e., *A. gossypii*, *Myzus persicae*, and *Melanaphis sacchari*) were scattered across the phylogenetic tree, indicating rapid evolution of *JHMAT*s in these species. In *A. gossypii*, *LOC114127483*, *LOC126549821*, and *LOC114129169* share higher sequence identity with each other than with *LOC114124070* (Figure 4B).

### 3.5 Silencing of *JHAMT1* disrupts wing differentiation of *A. gossypii*

**FIGURE 5:**
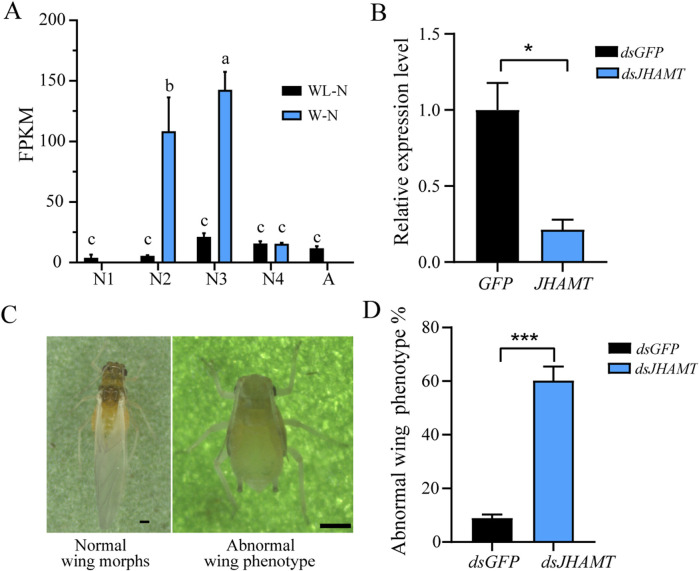
Validation of the role of *JHAMT* (LOC114124070) in the wing differentiation of *A. gossypii*. **(A)** Expression level of *LOC114124070* at different developmental stages of apterous and alate *A. gossypii*. WL–N = wingless morph, W–N = wing morph. N1, N2, N3, N4, and A represent the first-, second-, third-, and fourth-instar nymph and the adult, respectively. Different letters indicate significant difference at *p* < 0.05. **(B)** Relative expression of *JHMAT* (*LOC114124070*) after gene silencing. **(C)** Phenotypes of *A. gossypii* following *LOC114124070* silencing. **(D)** Phenotypic ratio of abnormal alate morphs in adults after *JHAMT-*silencing. Independent samples t-test was used for statistical analysis, and asterisks indicate significant differences at *p* < 0.001.

The expression level of *JHAMT* (LOC114124070) in the alate and apterous morphs across development exhibited two distinct peaks at the second- and third-instar nymphs of alate morphs (i.e., wing primordium and wing-sac formation stages, respectively). In contrast, no significant difference in *JHAMT* expression was observed across all the developmental stages of the apterous morph (Figure 5A). To assess the role of LOC114124070 in alate morph formation, corresponding dsRNA was administered to the third-instar alate nymphs via the ingestion method.

After 48-h treatment, *JHAMT1* expression in the treated group was significantly reduced by 79% compared with that in the control group (Figure 5B). Upon silencing of *JHAMT1*, numerous adult individuals exhibited abnormal phenotypes with degenerated wing buds instead of intact wings (Figure 5C), accounting for 60.2%—a proportion significantly higher than that in the control group (Figure 5D).

Notably, since only the silencing of *JHAMT1* was utilized in this study, precise regulation of JH titers in *A. gossypii* was not feasible. The third-instar duration of the dsGFP control group was approximately 1 day. Most *JHAMT1*-silenced individuals (dsJHAMT1 group) molted into normal fourth-instar nymphs with a delay of 1 to 2 days compared to those in the dsGFP group. No individuals in the dsJHAMT1 group exhibited precocious molting—i.e., direct development from the third-instar stage to adulthood or formation of larval–adult intermediates.

Given the complexity and diversity of adult abnormal phenotypes, only adult individuals with fully developed wings were recorded as normal, and the reduction in their proportion following *JHAMT1* inhibition was quantified; representative adult phenotypes are presented herein. The mortality rates of the ds*GFP* and ds*JHAMT1* groups were approximately equal (both <10%). The proportion of normally developed adults in both groups was calculated as 100% minus the abnormal ratio (Figure 5D).

## 4 Discussion

To clarify the wing differentiation dynamics of alate morphs in *A. gossypii*, newly born nymphs were used to induce alate morphs under high population density following the protocol described previously (Ji et al., 2019). Wing morph formation proceeds through four sequential stages, namely, wing primordia differentiation (second-instar nymph), wing capsule formation (third-instar nymph), wing bud formation (fourth-instar nymph), and full wing expansion (adult nymph) (Figure 1A), which is consistent with wing formation in male *A. gossypii* and *Megoura viciae* (Huangfu et al., 2022; Ganassi et al., 2005). Typically, wing morphs are induced by prenatal crowding, wherein mother aphids under high population density transmit signals to the offspring developing in their ovaries. Once born, a nymph’s developmental trajectory toward alate or apterous morph is set (Vellichirammal et al., 2017). Notably, in the present study, newly born nymphs could also differentiate into apterous or alate adults in response to postnatal population density (Figure 1A). This postnatal crowding-induced wing morph plasticity in newborn *A. gossypii* nymphs has also been observed in other aphid species such as *A. fabae*, *M. persicae*, *Therioaphis maculata*, and *Sitobion fragariae* (Müller et al., 2001). These results indicate that the early nymphal stage serves as a developmental switch point for wing determination in aphids.

Compared to the apterous morph, the number of upregulated and downregulated DEGs both gradually increased in alate nymphs, peaking at the fourth-instar stage, before decreasing sharply in adulthood (Figure 1C). Correspondingly, morphological differences between alate and apterous morphs were minimal before the fourth-instar nymphal stage, after which distinguishable wing buds protrude laterally from the aphid body (Figure 1; Supplementary Figure S2). Taken together, we speculate that genes highly expressed at the fourth-instar nymphal stage likely contribute significantly to wing bud formation and eclosion in the alate morph. In addition, upregulated DEGs at this stage were mainly enriched in pathways such as lysosome, galactose metabolism, amino acid biosynthesis, and insect hormone biosynthesis (Figure 2). This is consistent with a previous report showing that lipid metabolism-related genes are more highly expressed in alate morphs than in apterous morphs, indicating that energy allocation is crucial for winged metamorphosis (flight ability) in aphids (Yang et al., 2014). Therefore, these synergistic pathways that promote wing bud formation and differentiation warrant further investigation.

Interestingly, compared to the wingless morph, the upregulated DEGs of the winged morph from the second instar to adult stages were both enriched in the pathway of insect hormone (especially JH) biosynthesis (Figure 2A; Supplementary Table S1). JH signaling was considered to contribute to the growth and differentiation of insect wing imaginal disks (Chen H. et al., 2025). In *Nilaparvata lugens*, the decrease in JH significantly induced and increased long-wing proportion, whereas topical application of JHs or an analog (methoprene) to female nymphs near the penultimate instar significantly increased the proportion of brachypters (Chen R. et al., 2025; Iwanaga and Tojo, 1986). Studies in crickets have reported ambiguous results regarding whether JH, through regulation of trait expression, causes the short-winged morph (Zera, 2016). All these research studies indicate the importance of JH signaling in wing plasticity of insects.

Moreover, in contrast to the nearly constant expression level throughout the development of the apterous morph, *JHAMT* (*LOC114124070*)—a key regulatory enzyme in JH biosynthesis—exhibited significantly elevated transcriptional levels during the second- and third-instar stages. RNAi-mediated silencing of *JHAMT1* in alate morph nymphs resulted in wing bud degeneration in 60.2% of adults (Figure 5), consistent with findings in the red flour beetle. Simultaneous RNAi knockdown of *CYP15A1* (another key JH regulatory enzyme in JH biosynthesis) and *JHAMT* during the final larval stages of *T. castaneum* led to larval wing shortening, indicating the indispensability of JH signaling for wing expansion (Minakuchi et al., 2015). In contrast, dsRNA-mediated silencing of *JHEH* (a key juvenile hormone epoxide hydrolase involved in JH degradation) in the brown plant-hopper (*Nilaparvata lugens*) enhanced short-wing formation in the macropterous strain (Zhao et al., 2017), highlighting the complexity of JH signaling in insect wing differentiation.

Nevertheless, the role of *JHAMT* in regulating the development, metamorphosis, and reproduction of insects is well-documented. For example, knocking down *JHAMT* reduced JH titers and led to precocious nymphal ecdysis, metamorphosis, and impaired vitellogenesis in the migratory locust (Song et al., 2024). The inhibition of JHAMT enzyme activity through JHAMT-targeting inhibitor (JI3) can effectively disrupt the development and metamorphosis processes in *T. castaneum* (Yang et al., 2026). Knockdown of *JHAMT* induces diapause traits, including arrested ovarian development and increased lipid accumulation, in the bean bug *Riptortus pedestris* (Hafeez et al., 2025). In addition, the JHAMT^−/−^ larvae lacking JH died at the onset of metamorphosis (Nouzova et al., 2021). These studies provide an excellent paradigm for further investigating the molecular mechanisms by which *JHAMT* regulates wing differentiation and for identifying its upstream and downstream regulatory factors.

Our findings were supported by a study on the aphid *Megoura*, which showed short-winged morphology is produced by elevated levels of JH above a certain threshold, and *vice versa* (Zhang et al., 2019). However, it was found that in pea aphids, there was no correlation between maternal JH titers and the production of winged offspring (Schwartzberg et al., 2008). Therefore, the mechanism of how the *JHAMT* gene affects wing differentiation and formation in cotton aphids remains to be further explored. The dynamics of JH titers, the expression patterns of core response genes (*Met* and *Kr-h1*) in the JH signaling pathways, and rescue experiments with natural JH or a JH mimic after *JHAMT*1 silencing need to be examined in future studies. In addition, depleting other genes in JH signaling (*Gce*/*Met* ortholog, the three other *JHAMT*s, etc.) or biosynthesis (*CYP15C1* or *CYP15A1* ortholog), localization of *JHAMT*1 in corpora allata of *A. gossypii*, or RNAi in the first and second-instar juveniles of the winged morph needs further assessment.

## Data Availability

The data presented in the study are deposited in the NCBI repository, accession number PRJNA1406618.

## References

[B1] Braendle C. Davis G. K. Brisson J. A. Stern D. L. (2006). Wing dimorphism in aphids. Heredity 97, 192–199. 10.1038/sj.hdy.6800863 16823401

[B2] Brisson J. A. (2010). Aphid wing dimorphisms: linking environmental and genetic control of trait variation. Philosophical Trans. R. Soc. B-Biological Sci. 365, 605–616. 10.1098/rstb.2009.0255 20083636 PMC2817143

[B3] Buchfink B. Xie C. Huson D. H. (2015). Fast and sensitive protein alignment using DIAMOND. Nat. Methods 12, 59–60. 10.1038/nmeth.3176 25402007

[B4] Chen H. Liu Q. Xia Q. Zhao P. (2025a). Molecular basis and regulatory network of wing development in *Bombyx mori* . Insect Sci. 10.1111/1744-7917.70131 40874390

[B5] Chen R. Zhang H. Zou J. Chen J. Liu Z. (2025b). Nitenpyram resistance and IR56 feeding jointly drive the wing dimorphism in Nilaparvata lugens. J. Pest Sci. 98, 1905–1915. 10.1007/s10340-025-01911-4

[B6] Cheng S. H. Li R. Chen Z. Ni J. Lv N. Liang P. (2023). Comparative susceptibility of Aphis gossypii Glover (Hemiptera: aphididae) on cotton crops to imidacloprid and a novel insecticide cyproflanilide in China. Industrial Crops Prod. 192, 116053. 10.1016/j.indcrop.2022.116053

[B7] Eid A. E. El-Heneidy A. H. Hafez A. A. Shalaby F. F. Adly D. (2018). On the control of the cotton aphid, *Aphis gossypii* Glov. (Hemiptera: aphididae), on cucumber in greenhouses. Egypt. J. Biol. Pest Control 28 (1), 456–461. 10.1186/s41938-018-0065-9

[B8] Elbanhawy A. A. Elsherbiny E. A. Abd El-Mageed A. E. Abdel-Fattah G. M. (2019). Potential of fungal metabolites as a biocontrol agent against cotton aphid, *Aphis gossypii* Glover and the possible mechanisms of action. Pestic. Biochem. Physiol. 159, 34–40. 10.1016/j.pestbp.2019.05.013 31400782

[B9] Ganassi S. Signa G. Mola L. (2005). Development of the wing buds in Megoura viciae: a morphological study. Bull. Insectology 58, 101–105.

[B11] Guo H. J. Zhang Y. Li B. Li C. Shi Q. Zhu-Salzman K. (2023). Salivary carbonic anhydrase II in winged aphid morph facilitates infection viruses. Proc. Natl. Acad. Sci. U. S. A. 120, e2222040120. 10.1073/pnas.2222040120 36976769 PMC10083582

[B12] Hafeez A. Wang K. Liu W. Wang X. P. (2025). Juvenile hormone regulates reproductive diapause through both canonical and noncanonical pathways in the bean bug Riptortus pedestris. Insect Biochem. Mol. Biol. 177, 104233. 10.1016/j.ibmb.2024.104233 39622304

[B13] Heilsnis B. Mahas J. B. Conner K. Pandey S. Clark W. Koebernick J. (2023). Characterizing the vector competence of *Aphis gossypii, Myzus persicae* and *Aphis craccivora* (Hemiptera: aphididae) to transmit cotton leafroll dwarf virus to cotton in the United States. J. Econ. Entomol. 116, 719–725. 10.1093/jee/toad080 37171119 PMC10263271

[B14] Huangfu N. Shi Q. Chen L. Ma X. Zhang K. Li D. (2022). Comparative transcriptional analysis and identification of hub genes associated with wing differentiation of male in *Aphis gossypii* . J. Cotton Res. 5, 22. 10.1186/s42397-022-00130-x

[B15] Ishikawa A. Gotoh H. Abe T. Miura T. (2013). Juvenile hormone titer and wing-morph differentiation in the vetch aphid *Megoura crassicauda* . J. Insect Physiol. 59, 444–449. 10.1016/j.jinsphys.2013.02.004 23434762

[B16] Iwanaga K. Tojo S. (1986). Effects of juvenile hormone and rearing density on wing dimorphism and oöcyte development in the brown planthopper, *Nilaparvata lugens* . J. Insect Physiol. 32, 585–590. 10.1016/0022-1910(86)90076-4

[B17] Ji J. Zhang S. Luo J. Wang L. Zhu X. Zhang K. (2019). Comparative transcriptional analysis provides insights of possible molecular mechanisms of wing polyphenism induced by postnatal crowding in *Aphis gossypii* . J. Cotton Res. 2, 17. 10.1186/s42397-019-0036-z

[B18] Ji J. Huangfu N. Luo J. Gao X. Niu L. Zhang S. (2021). Insights into wing dimorphism in worldwide agricultural pest and host-alternating aphid Aphis gossypii. J. Cotton Res. 4, 5. 10.1186/s42397-021-00080-w

[B19] Jones P. Binns D. Chang H. Y. Fraser M. Li W. McAnulla C. (2014). InterProScan 5: genome-scale protein function classification. Bioinformatics 30, 1236–1240. 10.1093/bioinformatics/btu031 24451626 PMC3998142

[B20] Kim D. Landmead B. Salzberg S. L. (2015). HISAT: a fast spliced aligner with low memory requirements. Nat. Methods 12, 357–421. 10.1038/nmeth.3317 25751142 PMC4655817

[B21] Li R. Cheng S. Chen Z. Guo T. Liang P. Zhen C. (2022). Establishment of toxicity and susceptibility baseline of Broflanilide for *Aphis gossypii* Glove. Insects 13, 1033. 10.3390/insects13111033 36354856 PMC9695941

[B22] Li H. Kong X. Fang Y. Hou J. Zhang W. Zhang Y. (2025). Aphis craccivora (Hemiptera: aphididae) synthesizes juvenile hormone III *via* a pathway involving epoxidation followed by esterification, potentially providing an epoxidation active site for the synthesis of juvenile hormone SB3. Insect Sci. 32, 1311–1330. 10.1111/1744-7917.13450 39365891

[B23] Livak K. J. Schmittgen T. D. (2001). Analysis of relative gene expression data using real-time quantitative PCR and the 2^− ΔΔCT^ method. Methods 25, 402–408. 10.1006/meth.2001.1262 11846609

[B24] Ma K. S. Li F. Liang P. Z. Chen X. W. Liu Y. Gao X. W. (2016). Identification and validation of reference genes for the normalization of gene expression data in qRT-PCR analysis in *Aphis gossypii* (Hemiptera: aphididae). J. Insect Sci. 16. 10.1093/jisesa/iew003 28076279 PMC5778981

[B25] Minakuchi C. Ishii F. Washidu Y. Ichikawa A. Tanaka T. Miura K. (2015). Expressional and functional analysis of CYP15A1, a juvenile hormone epoxidase, in the red flour beetle *Tribolium castaneum* . J. Insect Physiol. 80, 61–70. 10.1016/j.jinsphys.2015.04.008 25921675

[B26] Müller C. B. Williams I. S. Hardie J. (2001). The role of nutrition, crowding and interspecific interactions in the development of winged aphids. Ecol. Entomol. 26, 330–340. 10.1046/j.1365-2311.2001.00321.x

[B27] Nouzova M. Edwards M. J. Michalkova V. Ramirez C. E. Ruiz M. Areiza M. (2021). Epoxidation of juvenile hormone was a key innovation improving insect reproductive fitness. Proc. Natl. Acad. Sci. U. S. A. 118, e2109381118. 10.1073/pnas.2109381118 34697248 PMC8609300

[B28] Ogawa K. Miura T. (2014). Aphid polyphenisms: trans-generational developmental regulation through viviparity. Front. Physiology 5, 1. 10.3389/fphys.2014.00001 24478714 PMC3900772

[B29] Pertea M. Pertea G. M. Antonescu C. M. Chang T. C. Mendell J. T. Salzberg S. L. (2015). StringTie enables improved reconstruction of a transcriptome from RNA-seq reads. Nat. Biotechnol. 33, 290–295. 10.1038/nbt.3122 25690850 PMC4643835

[B30] Schwartzberg E. G. Kunert G. Westerlund S. A. Hoffmann K. H. Weisser W. W. (2008). Juvenile hormone titres and winged offspring production do not correlate in the pea aphid, *Acyrthosiphon pisum* . J. Insect Physiol. 54, 1332–1336. 10.1016/j.jinsphys.2008.04.025 18634797

[B31] Shang F. Niu J. Ding B. Y. Zhang W. Wei D. D. Wei D. (2020). The miR-9b microRNA mediates dimorphism and development of wing in aphids. Proc. Natl. Acad. Sci. U. S. A. 117, 8404–8409. 10.1073/pnas.1919204117 32217736 PMC7165449

[B32] Shi D. Wang T. Lv H. Li X. Wan H. He S. (2023). Insecticide resistance monitoring and diagnostics of resistance mechanisms in cotton-melon aphid. Aphis Gossypii Glover Central China 147, 392–405. 10.1111/jen.13119

[B33] Simon J. C. Peccoud J. (2018). Rapid evolution of aphid pests in agricultural environments. Curr. Opinion Insect Science 26, 17–24. 10.1016/j.cois.2017.12.009 29764656

[B34] Song J. Li W. Gao L. Yan Q. Zhang X. Liu M. (2024). miR-276 and miR-182013-5p modulate insect metamorphosis and reproduction *via* dually regulating juvenile hormone acid methyltransferase. Commun. Biol. 7, 1604. 10.1038/s42003-024-07285-0 39623057 PMC11612435

[B35] Song Y. X. Ren Y. P. Ran Y. Y. Fan N. Wu T. Zang H. (2026). Ame-miR-2161 affects the survival and development of honeybee larvae through the juvenile hormone acid methyltransferase gene. Insect Mol. Biol. 35, 79–90. 10.1111/imb.70009 40801905

[B36] Strassburger K. Lutz M. Müller S. Teleman A. A. (2021). Ecdysone regulates Drosophila wing disc size *via* a TORC1 dependent mechanism. Nat. Commun. 12, 6684. 10.1038/s41467-021-26780-0 34795214 PMC8602387

[B37] Tsuji H. Kawada K. (1987). Development and degeneration of wing buds and indirect flight muscles in the pea aphid (*Acyrthosiphon pisum* (Harris)). Jpn. J. Appl. Entomology Zoology 31, 247–252. 10.1303/jjaez.31.247

[B38] Tsumuki H. Kawada K. Nakehisa K. Nagatsuka H. (1990). Comparison of nutrient reseevation in apterous and alate pea aphids, *Acyrthosiphon pisum* (harris): 2. Amino Acid contents. Appl. Entomol. Zool. 25, 223–229. 10.1303/aez.25.223

[B39] Vellichirammal N. N. Gupta P. Hall T. A. Brisson J. A. (2017). Ecdysone signaling underlies the pea aphid transgenerational wing polyphenism. Proc. Natl. Acad. Sci. U. S. A. 114, 1419–1423. 10.1073/pnas.1617640114 28115695 PMC5307454

[B40] Wang L. Y. Hui C. Sandhu H. S. Li Z. H. Zhao Z. H. (2015). Population dynamics and associated factors of cereal aphids and armyworms under global change. Sci. Rep. 5, 18801. 10.1038/srep18801 26689373 PMC4686941

[B41] Wills W. K. (2017). State of the world’s plants. Royal Botanic Gardens Kew. 29144713

[B42] Yang X. Liu X. Xu X. Li Z. Li Y. Song D. (2014). Gene expression profiling in winged and wingless cotton aphids, *Aphis gossypii* (Hemiptera: aphididae). Int. J. Biol. Sci. 10, 257–267. 10.7150/ijbs.7629 24644424 PMC3957081

[B43] Yang Y. Li Q. Zheng H. Shi J. Hu Y. Xia Y. (2026). Unveiling the molecular architecture of TcJHAMT: structural basis for catalytic efficiency and targeted inhibitor screening in the red flour beetle, Tribolium castaneum. Pest Manag. Sci. 82, 1090–1099. 10.1002/ps.70267 41108193

[B44] Yuan E. Guo H. Chen W. Du B. Mi Y. Qi Z. (2023). A novel gene REPTOR2 activates the autophagic degradation of wing disc in pea aphid. eLife 12, e83023. 10.7554/eLife.83023 36943031 PMC10030113

[B45] Zera A. J. (2016). Juvenile Hormone and the endocrine regulation of wing polymorphism in insects: new insights from circadian and functional-genomic studies in Gryllus crickets. Physiol. Entomol. 41, 313–326. 10.1111/phen.12166

[B46] Zhang C. X. Brisson J. A. Xu H. J. (2019). Annual review of entomology. Mole. Mech. Wing Polymorph. Insects 64, 297–314. 10.1146/annurev-ento-011118-112448 30312555

[B47] Zhang S. Gao X. Wang L. Jiang W. Su H. Jing T. (2022). Chromosome-level genome assemblies of two cotton-melon aphid *Aphis gossypii* biotypes unveil mechanisms of host adaption. Mol. Ecol. Resour. 22, 1120–1134. 10.1111/1755-0998.13521 34601821

[B48] Zhao J. Zhou Y. Li X. Cai W. Hua H. (2017). Silencing of juvenile hormone epoxide hydrolase gene (Nljheh) enhances short wing formation in a macropterous strain of the brown planthopper, *Nilaparvata lugens* . J. Insect Physiol. 102, 18–26. 10.1016/j.jinsphys.2017.08.012 28867330

