# Supplementary figures and images for: The JHAMT1 gene is necessary for wing differentiation in Aphis gossypii
Source: Front Physiol. 2026 Mar 10;17:1747260. doi: 10.3389/fphys.2026.1747260 (PMC13008626; doi:10.3389/fphys.2026.1747260)

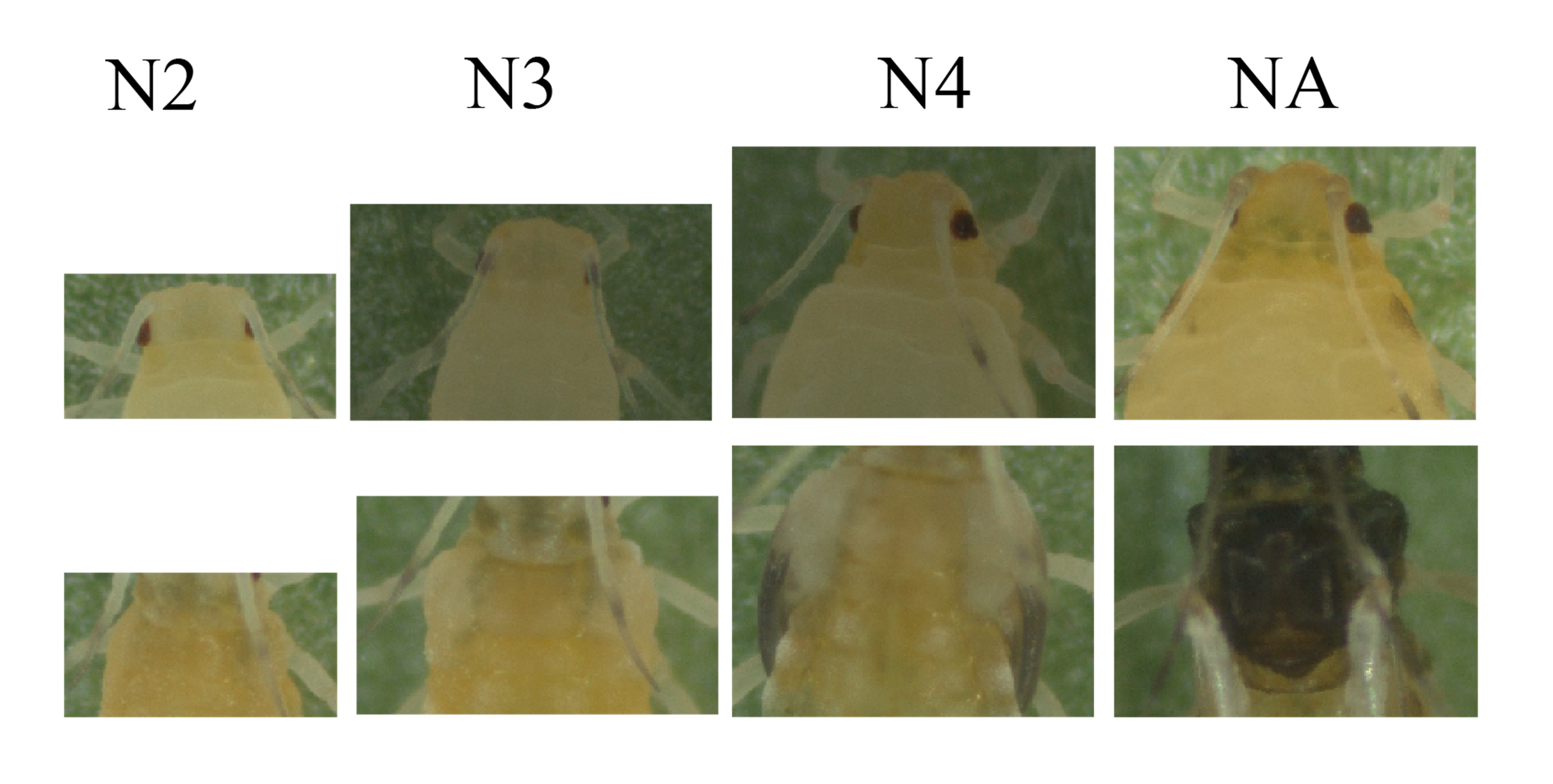

Supplement: Supplementary file 3 [file Image2.tif]

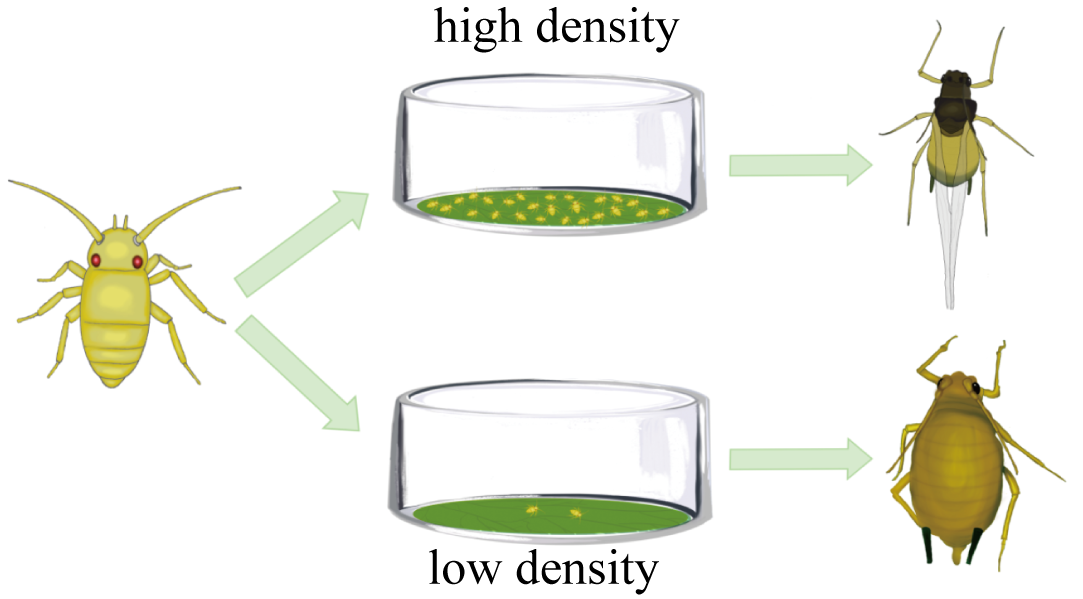

Supplement: Supplementary file 4 [file Image1.tif]
